# Zebrin II Expressing Purkinje Cell Phenotype—Related and—Unrelated Cerebellar Abnormalities in Ca_˅_2.1 Mutant, Rolling Mouse Nagoya

**DOI:** 10.1100/tsw.2010.205

**Published:** 2010-10-12

**Authors:** Kazuhiko Sawada, Yoshihiro Fukui

**Affiliations:** ^1^ Laboratory of Anatomy, Department of Physical Therapy, Faculty of Medical and Health Sciences, Tsukuba International University, Tsuchiura, Ibaraki, Japan; ^2^ Department of Anatomy and Developmental Neurobiology, University of Tokushima Graduate School, Institute of Health Biosciences, Tokushima, Japan

**Keywords:** Ca^2+^ channelopathy, zebrin, tyrosine hydroxylase, ryanodine receptor, CRF, serotonin

## Abstract

Rolling mouse Nagoya is an ataxic mutant mouse that carries a mutation in a gene encoding for the alpha 1A subunit of the voltage-gated P/Q-type Ca^2+^ channel (Ca_˅_2.1). This report summarizes our studies and others concerning cerebellar abnormalities in rolling mice based on chemical neuroanatomy. While there are no obvious cerebellar deformations in this mutant mouse, the altered functions of Purkinje cells can be revealed as a reduced expression of type 1 ryanodine receptor (RyR1) in all Purkinje cells uniformly throughout the cerebellum, and as an ectopic expression of tyrosine hydroxylase (TH) in the Purkinje cell subsets with the zebrin II—immunopositive phenotype. As the mutated Ca_˅_2.1 channel is expressed at uniform levels in all Purkinje cells, its copresence with RyR1 staining suggests that a Ca_˅_2.1 channel dysfunction links with the expression of RyR1 in Purkinje cells of rolling mice. However, an ectopic expression of TH in the Purkinje cells is topologically related to the projection of corticotrophin-releasing factor—immunopositive climbing fibers rather than expression of the mutated Ca_˅_2.1 channel. On the other hand, increased levels of serotonin (5-HT) in 5-HTergic fibers were revealed immunohistochemically in Purkinje cells of the vermis of rolling cerebellum. Thus, to determine whether or not cerebellar abnormalities are related to Purkinje cell populations revealed by zebrin II expression is essential for enhancing our understanding of the pathogenesis of hereditary cerebellar ataxic mutants such as rolling mice.

## INTRODUCTION

Rolling mouse Nagoya is an ataxic mutant mouse that carries a mutation in a gene encoding for the $\alpha_{1A}$ subunit of the voltage-gated P/Q-type $Ca^{2+}$ channels ($Ca_v2.1$)[1], as do tottering, leaner[2], rocker[3], and wobbly mice[4]. Specifically, nucleotide position 3784 of the $Ca_v2.1$ gene is changed from C to G, resulting in a charge-neutralizing amino acid change from a highly conserved arginine to glycine at position 1262 in the voltage sensor-forming segment of the $Ca_v2.1$ channel protein[1]. By this mutation, the voltage sensitivity and the activity of P/Q-type $Ca_v2.1$ channel are selectively reduced in cerebellar Purkinje cells[1]. In humans, defects in this gene are responsible for several neurological disorders, such as episodic ataxia type-2 and spinocerebellar ataxia type-6[5]. Motor deficits of rolling mice are characterized by frequent lurching of the mice and abnormal cyclic movements of the hindlimbs when walking, but they do not exhibit epilepsy as seen in tottering and leaner mice[6,7]. However, no obvious cerebellar deformations are seen in rolling mice[8]. As the $Ca_v2.1$ channel gene mutation selectively reduces the voltage sensitivity and activity of this channel[1,2,9], $Ca_v2.1$ channel dysfunctions are considered to be fundamentally attributable to the motor deficits of rolling mice[8]. However, the underlying pathogenesis associated with or resulting from this mutation is not easy to understand because a complex relationship between $Ca_v2.1$ expression and cerebellar abnormalities was already revealed in our previous studies and those of others. The Purkinje cells of the cerebellar cortex form a complex arrangement of parasagittal stripes and transverse zones, which are reflected in the diversity of gene expression patterns, such as zebrin II, phospholipase Cß4, and the small heat shock protein HSP25[10,11,12,13,14]. This report summarizes our and other studies regarding cerebellar abnormalities either related or unrelated to specific Purkinje cell populations revealed by zebrin II expression in rolling mice.

## EXPRESSION OF MUTATED $CA_V2.1$ CHANNELS IN ROLLING MOUSE CEREBELLUM

The mutated $Ca_v2.1$ channel is expressed uniformly in all Purkinje cells in rolling[8,15] and other $Ca_v2.1$ mutant mice[2,16]. The $Ca_v2.1$ channel gene mutation selectively reduces the voltage sensitivity and activity of this channel[1,2,9]. Such dysfunctions of the $Ca_v2.1$ channel alter Purkinje cell morphology. Electronmicroscopic observations revealed multiple synapses between the dendritic spines of Purkinje cells at single parallel fiber varicosity in rolling mice[17] and other $Ca_v2.1$ mutants[18]. Furthermore, abnormal swellings (or “torpedoes”), which are known to be a neuropathological sign of axonal transport impairments[19], were seen in Purkinje cell axons of rolling mice (Fig. 1A, 1A’)[8] as well as in other $Ca_v2.1$ mutant mice[20].

## TYPE 1 RYANODINE RECEPTOR EXPRESSION

Our recent study revealed that type 1 ryanodine receptor (RyR1) expression in Purkinje cells was uniformly reduced through all cerebellar lobules in rolling mice[21]. Such an expressional change was not regionally specific or related to any Purkinje cell phenotypes. Coexpression of RyR1 and the $Ca_v2.1$ channel in all Purkinje cells of rolling mice indicates that expressional changes in RyR1are linked with the $Ca_v2.1$ channel dysfunction[21]. RyR1 expression also overlapped with the L-type $Ca^{2+}$ channel $\alpha_{1C}$ subunit ($Ca_v1.3$) in the cerebellum of rolling mice[21]. The $Ca_v1.3$ channel is known as the dihydropyridine receptor (DHPR) and has a direct conformational linkage with RyR1 in the neuronal plasma membrane to induce a $Ca^{2+}$ release from the ER through RyR1 (DHPR-induced $Ca^{2+}$ release)[22]. Therefore, DHPR-RyR1 complexes in Purkinje cells of rolling mice may be reduced by a diminished RyR1 expression.

## TYROSINE HYDROXYLASE EXPRESSION

Tyrosine hydroxylase (TH), the first-step enzyme for catecholamine synthesis, is mainly expressed in catecholaminergic neurons in the brain, but has also been expressed in some noncatecholaminergic neurons

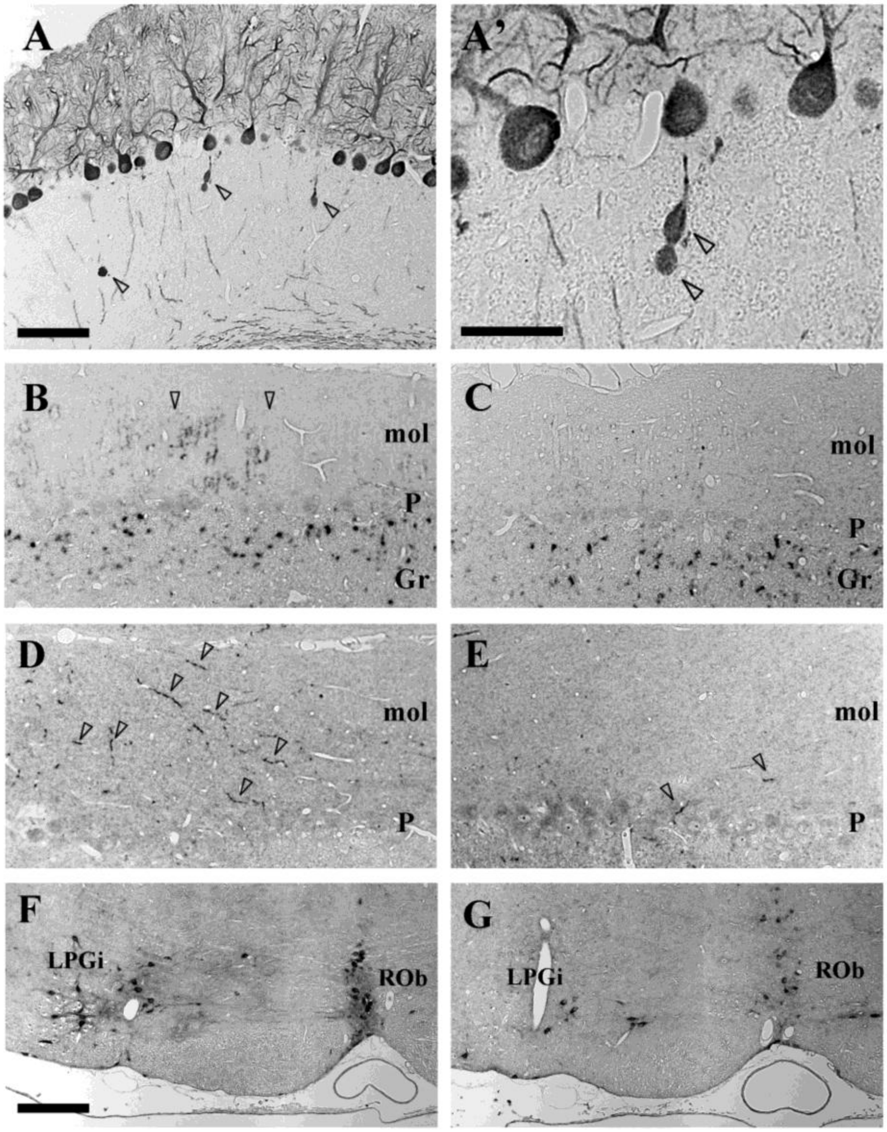

**FIGURE 1.** Cerebellar abnormalities of rolling mouse Nagoya. Calbindin D-28k immunostaining in the cerebellum of rolling mouse at low (A) and high (A') magnifications. Arrowheads indicate abnormal swellings (torpedoes) of Purkinje cell axons. Immunostaining for corticotropin-releasing factor (CRF) in the cerebellum of rolling (B) and control mice (C). Arrowheads in (B) indicate the borders of CRF-immunopositive climbing fiber stripe. The staining intensity is enhanced in the rolling cerebellum, whereas the characteristic stripe pattern of CRF is sustained between rolling and control mice. Serotonin (5-HT) immunostaining in the cerebellar cortex of the vermis (D, E) and the origins of cerebellar 5-HTergic fibers (F, G). 5-HT–immunopositive fibers were densely present in the vermis of a rolling mouse (D), but not a control mouse (E). Arrowheads in (D) and (E) indicate 5-HT–immunopositive fibers. 5-HT immunostaining in the lateral paragigantocellular reticular nucleus of medulla oblongata was also more intense in a rolling mouse (F) than in a control mouse (G). LPGi, lateral paragigantocellular reticular nucleus; mol, molecular layer; P, Purkinje cell layer; ROb, raphe obscurus. Bar = 50 μm in (A)[applied to (B), (C), (D), and (E)], Bar = 20 μm in (A'), Bar = 100 μm in (F)[applied to (G)].

of various brain regions during development and in experimental conditions, such as organotypic tissue culture and transplantation[23]. We have reported that TH was ectopically expressed in particular subsets of Purkinje cells in the rolling mouse cerebellum[23,24]. Ectopic expression of TH in the Purkinje cells is not specific to the $Ca_v2.1$ mutant[23,24], but is rather thought to reflect an increase of intracellular $Ca^{2+}$

concentrations[23]. Furthermore, the enzymatically active, phosphorylated form of TH has not been identified immunohistochemically in the Purkinje cells of rolling mice[25], suggesting the absence of any aberrant catecholamine synthesis. Interestingly, the expression pattern of TH resembles that of zebrin II[24], which is known as the most extensively studied marker of Purkinje cell phenotypes[26,27]. However, neither of those two distributions completely overlapped; TH-immunopositive Purkinje stripes were also present in the central zone (lobules VI to VII) and nodular zone (ventral IX and lobule X), where zebrin II is uniformly expressed[24]. TH-immunopositive Purkinje cell stripes in these regions formed an alternating array to HSP25-immunopositive Purkinje cell stripes[28]. As described in the next section, such ectopic TH expression in the rolling Purkinje cells is considered to be associated with corticotropin-releasing factor (CRF)–immunopositive climbing fiber innervation and not with expression of the mutated $Ca_v$ 2.1 channel.

## INNERVATION OF CRF-IMMUNOPOSITIVE CLIMBING FIBERS

CRF immunostaining is found in climbing fiber subsets in the cerebellum, and a striking parasagittal pattern of CRF-immunopositive climbing fibers has been reported in several mammalian species[29,30,31,32,33,34]. In normal mice, the array of CRF-immunopositive climbing fiber stripes resembles the zebrin II–immunopositive Purkinje cell stripes, whereas these two distributions did not completely overlap[34]. CRF immunostaining in particular subsets of climbing fibers is enhanced in the rolling cerebellum (Fig. 1B,C)[35]. Our previous studies revealed that the overall distribution of CRF-immunopositive climbing fibers was not altered[35]. Furthermore, the expression of CRF mRNA increased in the inferior olivary origin of the CRF-immunopositive climbing fibers in rolling mice[36]. It is interesting to note that TH is ectopically expressed in the rolling mouse cerebellum corresponding to the terminal fields of CRF-immunopositive climbing fibers[35]. This suggested that CRF from a particular component of olivocerebellar tracts is involved in ectopic TH expression in the Purkinje cells of rolling mice. While the $Ca_v$ 2.1 channel is strongly expressed in the inferior olivary nucleus[37], the relationship is unclear between the $Ca_v$ 2.1 channel function and CRF expression in this nucleus.

On the other hand, CRF serves as a neuromodulator in the cerebellar circuit to enhance the glutamate sensitivity and reduce the GABA sensitivity of Purkinje cells[38,39] by suppressing the afterhyperpolarization[40]. Furthermore, CRF from climbing fibers plays a critical role in the induction of long-term depression (LTD) at the parallel fiber synapses of Purkinje cells[41]. Therefore, increased levels of CRF in climbing fibers of the rolling mouse cerebellum may alter the excitability and/or LTD induction of zebrin II–like, TH-defined Purkinje cell populations.

## SEROTONERGIC INNERVATION

The enhancement of the serotonin (5-HT) metabolism and an increased 5-HTergic innervation have been revealed in the cerebellum of other ataxic mutant mice[42,43]. 5-HTergic fibers are distributed throughout the cerebellar cortex[44,45], and reduce Purkinje cell activity either directly or via Lugaro cells[46,47]. The distribution of 5-HT–immunopositive fibers in the vermis of the cerebellar cortex was much denser in rolling mice than in wild-type mice (Fig. 1D, E). However, 5-HT–immunopositive fibers were few in the hemisphere in both rolling and control mice. In addition, the distribution of 5-TH–immunopositive fibers in the rolling cerebellum did not involve either zebrin II– or TH-defined Purkinje cell compartments.

5-HT immunostaining of the origins of cerebellar 5-HTergic fibers (the lateral paragigantocellular reticular nucleus of medulla oblongata) was also enhanced in rolling mice (Fig. 1F, G). Since an enhancement of 5-HT concentration in both the cerebellum and brainstem has been reported in rolling mice[48], much denser 5-HT–immunopositive fibers in the rolling mouse than in the wild-type cerebellum may reflect increased levels of 5-HT in the cerebellar 5-HTergic fibers in the former. This suggests that such increased 5-HTergic innervations of the cerebellar cortex may alter the regulation of Purkinje cell excitability and/or of the LTD in rolling mice.

The $Ca_v2.1$ channel is expressed in the medullary raphe nuclei[37] and blockade of this channel is known to enhance the firing rate of medullary raphe neurons by reducing afterhyperpolarization[49]. Therefore, the increased 5-HTergic innervations of the rolling cerebellum may be involved in the enhanced excitability of medullary raphe neurons by dysfunction of the mutated $Ca_v2.1$ channel.

## CONCLUSIONS

Although motor deficits of rolling mice are characterized by frequent lurching of the mice and abnormal cyclic movements of the hindlimbs when walking[6,7], no obvious cerebellar deformations have been reported in this mutant. Our studies of the rolling mouse cerebellum have revealed the cerebellar abnormalities based on chemical neuroanatomy. The reduced RyR1 expressions in Purkinje cells and the increased 5-HTergic innervations of the cerebellar cortex in rolling mice may be linked with $Ca_v2.1$ channel expression[21]. Ectopic TH expression appeared within a specific Purkinje cell population revealed by zebrin II expression[23,24] and was related to the innervations of CRF-immunopositive climbing fibers[35], whereas the relationship between the $Ca_v2.1$ channel function and CRF expression of the inferior olivary nucleus was unclear. Thus, to determine whether or not cerebellar abnormalities are related to Purkinje cell populations revealed by zebrin II expression is essential for enhancing our understanding of the pathogenesis of hereditary cerebellar ataxic mutants such as rolling mice.

## ACKNOWLEDGMENTS

The authors would like to thank Dr. Oda, of the Graduate School of Bio-Agricultural Science, Nagoya University, for providing the rolling mice.

## References

1. Mori, Y., Wakamori, M., Oda, S., Fletcher, C.F., Sekiguchi, N., Mori, E., Copeland, N.G., Jenkins, N.A., Matsushita, K., Matsuyama, Z., and Imoto, K. (2000) Reduced voltage sensitivity and activation of P/Q-type $Ca^{2+}$ channels is associated with the ataxic mouse mutation rolling Nagoya (*tg*$^{rol}$). *J. Neurosci.* **20,** 5654–5662.
2. Fletcher, C.F., Lutz, C.M., O'Sullivan, T.N., Shaughnessy, J.D., Jr., Hawkes, R., Frankel, W.N., Copeland, N.G., and Jenkins, N.A. (1996) Absence epilepsy in *tottering* mutant mice is associated with calcium channel defects. *Cell* **87,** 607–617.
3. Zwingman, T.A., Neumann, P.E., Noebels, J.L., and Herrup, K. (2001) Rocker is a new variant of the voltage-dependent calcium channel gene *Cacna1a*. *J. Neurosci.* **21,** 1169–1178.
4. Xie, G., Clapcote, S.J., Nieman, B.J., Tallerico, T., Huang ,Y., Vukobradovic, I., Cordes, S.P., Osborne, L.R., Rossant, J., Sled, J.G., Henderson, J.T., and Roder, J.C. (2007) Forward genetic screen of mouse reveals dominant missense mutation in the P/Q-type voltage-dependent calcium channel, CACNA1A. *Genes Brain Behav.* **6,** 717–727.
5. Ophoff, R.A., Terwindt, G.M., Vergouwe, M.N., van Eijk, R., Oefner, P.J., Hoffman, S.M.G., Lamerdin, J.E., Mohrenweiser, H.W., Bulman, D.E., Ferrari, M., Haan, J., Lindhout, D., van Ommen, G.B., Hofker, M.H., Ferrari, M.D., and Frants, R.R. (1996) Familial hemiplegic migraine and episodic ataxia type-2 are caused by mutations in the $Ca^{2+}$ channel gene *CACNL1A4*. *Cell* **87,** 543–552.
6. Tamaki, Y., Oda, S., and Kameyama, Y. (1986) Postnatal locomotion development in a neurobiological mutant of rolling mouse Nagoya. *Dev. Psychobiol.* **19**, 67–77.
7. Plomp, J.J., van den Maagdenberg, A.M.J.M., and Kaja, S. (2009) The ataxic Cacna1a-mutant mouse rolling Nagoya: an overview of neuromorphological and electrophysiological findings. *Cerebellum* **8**, 222–230.
8. Sawada, K., Haga, H., and Fukui, Y. (2000) Ataxic mutant mice with defects in $Ca^{2+}$ channel $\alpha_{1A}$ subunit gene: morphological and functional abnormalities in cerebellar cortical neurons. *Congenit. Anom. Kyoto* **40,** 99–107.

9. Wakamori, M., Yamazaki, K., Matsunodaira, H., Teramoto, T., Tanaka, I., Niidome, T., Sawada, K., Nishizawa, Y., Sekiguchi, N., Mori, E., Mori, Y., and Imoto, K. (1998) Single tottering mutations responsible for the neuropathic phenotype of the P-type calcium channel. *J. Biol. Chem.* **273,** 34857–34867.
10. Hawkes, R. (1997) An anatomical model of cerebellar modules. *Prog. Brain Res.* **114,** 39–52.
11. Herrup, K. and Kuemerle, B. (1997) The compartmentalization of the cerebellum. *Annu. Rev. Neurosci.* **20,** 61–90.
12. Oberdick, J., Baader, S.L., and Schilling, K. (1998) From zebra stripes to postal zones: deciphering patterns of gene expression in the cerebellum. *Trends Neurosci.* **21,** 383–390.
13. Armstrong, C.L. and Hawkes, R. (2000) Pattern formation in the cerebellar cortex. *Biochem. Cell Biol.* **78,** 551–562.
14. Sarna, J.R., Marzban, H., Watanabe, M., and Hawkes, R. (2006) Complementary stripes of phospholipase Cß3 and Cß4 expression by Purkinje cell subsets in the mouse cerebellum. *J. Comp. Neurol.* **496,** 303–313.
15. Sawada, K., Sakata-Haga, H., Ando, M., Takeda, N., and Fukui, Y. (2001) An increased expression of $Ca^{2+}$ channel $\alpha_{1A}$ subunit immunoreactivity in deep cerebellar neurons of rolling mouse Nagoya. *Neurosci. Lett.* **316,** 87–90.
16. Lau, F.C., Abbott, L.C., Rhyu, I.J., Kim, D.S., and Chin, H. (1998) Expression of calcium channel $\alpha_{1A}$ mRNA and protein in the leaner mouse ($tg^{la}/tg^{la}$) cerebellum. *Mol. Brain Res.* **59,** 93–99.
17. Rhyu, I.J., Oda, S., Uhm, C.S., Kim, H., Suh, Y.S., and Abbott, L.C. (1999) Morphological investigation of *rolling* mouse Nagoya ($tg^{rol}/tg^{rol}$), cerebellar Purkinje cells: an ataxic mutant revisited. *Neurosci. Lett.* **266,** 49–52.
18. Rhyu, I.J., Abbott, L.C., Walker, D.B., and Sotelo, C. (1999) An ultrastructural study of granule cell/ Purkinje cell synapses in tottering (*tg/tg*), leaner ($tg^{la}/tg^{la}$) and compound heterozygous tottering/leaner (tg/tgla) mice. *Neuroscience* **90,** 717–728.
19. Carpenter, S., Karpati, G., Andermann, F., and Gold, R. (1974) Giant axonal neuropathy. *Arch. Neurol.* **31,** 312–316.
20. Abbott, L.C., Isaacs, K.R., and Heckroth, J.A. (1996) Co-localization of tyrosine hydroxylase and zebrin II immunoreactivities in Purkinje cells of the mutant mice, tottering and tottering/leaner. *Neuroscience* **714,** 461–475.
21. Sawada, K., Hosoi, E., Bando, M., Sakata-Haga, H., Lee, N.S., Jeong, Y.G., and Fukui, Y. (2008) Differential alterations in expressions of ryanodine receptor subtypes in cerebellar cortical neurons of an ataxic mutant, rolling mouse Nagoya. *Neuroscience* **152,** 609–617.
22. Mouton, J., Marty, I., Villaz, M., Feltz, A., and Maulet, Y. (2001) Molecular interaction of dihydropyridine receptors with type-1 ryanodine receptors in rat brain. *Biochem. J.* **354,** 597–603.
23. Sawada, K. and Fukui, Y. (2001) Expression of tyrosine hydroxylase in cerebellar Purkinje cells of ataxic mutant mice: its relation to the onset and/or development of ataxia. *J. Med. Invest.* **48,** 5–10.
24. Sawada, K., Komatsu, S., Haga, H., Sun, X.Z., Hisano, S., and Fukui, Y. (1999) Abnormal expression of tyrosine hydroxylase immunoreactivity in cerebellar cortex of ataxic mutant mice. *Brain Res.* **829,** 107–112.
25. Sawada, K., Ando, M., Sakata-Haga, H., Sun, X.Z., Jeong, Y.G., Hisano, S., Takeda, N., and Fukui, Y. (2004) Abnormal expression of tyrosine hydroxylase not accompanied by phosphorylation at serine 40 in cerebellar Purkinje cells of ataxic mutant mice, rolling mouse Nagoya and *dilute-lethal. Congenit. Anom. Kyoto* **44,** 46–50.
26. Brochu, G., Maler, L., and Hawkes, R. (1990) Zebrin II: a polypeptide antigen expressed selectively by Purkinje cells reveals compartments in rats and fish cerebellum. *J. Comp. Neurol.* **291,** 538–552.
27. Ahn, A.H., Dziennis, S., Hawkes, R., and Herrup, K. (1994) The cloning of zebrin II reveals its identity with aldolase C. *Development* **120,** 2081–2090.
28. Sawada, K., Sakata-Haga, H., and Fukui, Y. (2010) Alternating array of tyrosine hydroxylase and heat shock protein 25 immunopositive Purkinje cell stripes in zebrin II-defined transverse zone of the cerebellum of rolling mouse Nagoya. *Brain Res.* **1343,** 46–53.
29. Cha, C.I. and Foote, S.L. (1988) Corticotropin-releasing factor in olivocerebellar climbing fiber system of monkey (*Saimiri sciureus* and *Macaca fascicularis*): parasagittal and regional organization visualized by immunohistochemistry. *J. Neurosci.* **8,** 4121–4137.
30. Cummings, S., Sharp, B., and Elde, R. (1988) Corticotropin-releasing factor in cerebellar afferent systems: a combined immunohistochemistry and retrograde transport study. *J. Neurosci.* **8,** 543–554.
31. Cummings, S.L., Young, W.S., III, Bishop, G.A., DeSouza, E.B., and King, J.S. (1989) Distribution of corticotropin-releasing factor in the cerebellum and precerebellar nuclei of the opossum: a study utilizing immunohistochemistry, in situ hybridization histochemistry, and receptor autoradiography. *J. Comp. Neurol.* **280,** 501–521.
32. Errico, P. and Barmack, N.H. (1993) Origins of cerebellar mossy and climbing fibers positive for corticotropin-releasing factor in the rabbit. *J. Comp. Neurol.* **336,** 307–320.
33. King, J.S., Madtes, P., Jr., Bishop, G.A., and Overbeck, T.L. (1997) The distribution of corticotropin-releasing factor (CRF), CRF binding sites and $CRF_1$ receptor mRNA in the mouse cerebellum. *Prog. Brain Res.* **114,** 55–66.
34. Sawada, K., Fukui, Y., and Hawkes, R. (2008) Spatial distribution of corticotropin-releasing factor immunopositive climbing fibers in the mouse cerebellum: analysis by whole mount immunohistochemistry. *Brain Res.* **1222,** 106–117.
35. Sawada, K., Sakata-Haga, H., Hisano, S., and Fukui, Y. (2001) Topological relationship between corticotropin-releasing factor-immunoreactive cerebellar afferents and tyrosine hydroxylase-immunoreactive Purkinje cells in a hereditary ataxic mutant, rolling mouse Nagoya. *Neuroscience* **102,** 925–935.

36. Sawada, K., Kawano, M., Tsuji, H., Sakata-Haga, H., Hisano, S., and Fukui, Y. (2003) Over-expression of corticotropin-releasing factor mRNA in inferior olivary neurons of rolling mouse Nagoya. *Mol. Brain Res.* **117,** 190–195.
37. Craig, P.J., McAinsh, A.D., McCormack, A.L., Smith, W., Beattie, R.E., Priestley, J.V., Yip, J.L.Y., Averill, S., Longbottom, E.R., and Volsen, S.G. (1998) Distribution of the voltage-dependent calcium channel $\alpha_{1A}$ subunit throughout the mature rat brain and its relationship to neurotransmitter pathways. *J. Comp. Neurol.* **397,** 251–267.
38. Bishop, G.A. (1990) Neuromodulatory effects of corticotropin releasing factor on cerebellar Purkinje cells: an in vivo study in the cat. *Neuroscience* **39,** 251–257.
39. Bishop, G.A. and King J.S. (1992) Differential modulation of Purkinje cell activity by enkephalin and corticotropin releasing factor. *Neuropeptides* **22,** 167–174.
40. Fox, E.A. and Gruol, D.L. (1993) Corticotropin-releasing factor suppresses the afterhyperpolarization in cerebellar Purkinje neurons. *Neurosci. Lett.* **149,** 103–107.
41. Miyata, M., Okada, D., Hashimoto, K., Kano, M., and Ito, M. (1999) Corticotropin-releasing factor plays a permissive role in cerebellar long-term depression. *Neuron* **22,** 763–775.
42. Ohisugi, K., Adachi, K., and Ando, M. (1986) Serotonin metabolism in the CNS in cerebellar ataxic mice. *Experimentia* **42,** 1245–1247.
43. Triarhou, L.C. and Ghetti, B. (1991) Serotonin-immunoreactivity in the cerebellum of two neurological mutant mice and the corresponding wild-type genetic stocks. *J. Chem. Neuroanat.* **4,** 321–428.
44. Takeuchi, Y., Kimura, H., and Sano, Y. (1982) Immunohistochemical demonstration of serotonin-containing nerve fibers in the cerebellum. *Cell Tissue Res.* **226,** 1–12.
45. Bishop, G.A. and Ho, R.H. (1985) The distribution and origin of serotonin immunoreactivity in the rat cerebellum. *Brain Res.* **331,** 195–207.
46. Dean, I., Robertson, S.J., and Edwards, F.A. (2003) Serotonin drives a novel GABAergic synaptic current recorded in rat cerebellar Purkinje cells: a Lugaro cell to Purkinje cell synapse. *J. Neurosci*. **23,** 4457–4469.
47. Schweighofer, N., Doya, K., and Kuroda, S. (2004) Cerebellar aminergic neuromodulation: towards a functional understanding. *Brain Res. Rev.* **44,** 103–116.
48. Nakamura, T., Honda, M., Kimura, S., Tanabe, M., Oda, S., and Ono, H. (2005) Taltirelin improves motor ataxia independently of monoamine levels in rolling mouse Nagoya: a model of spinocerebellar atrophy. *Biol. Pharm. Bull.* **28,** 2244–2247.
49. Bayliss, D.A., Li, Y.-W., and Talley, E.M. (1997) Effects of serotonin on caudal raphe neurons: inhibition of N- and P/Q-type calcium channels and the afterhyperpolarization. *J. Neurophysiol.* **77,** 1362–1374.
